# T223. REAL WORLD EFFECTIVENESS OF ANTIPSYCHOTIC DRUGS IN PATIENTS WITH SCHIZOPHRENIA: A 10-YEARS RETROSPECTIVE STUDY

**DOI:** 10.1093/schbul/sby016.499

**Published:** 2018-04-01

**Authors:** Tae Young Lee, Minah Kim, Junhee Lee, Jun Soo Kwon

**Affiliations:** Seoul National University College of Medicine

## Abstract

**Background:**

Discontinuation of antipsychotics in patients with schizophrenia has increasing attention as a representative treatment effectiveness of the medication. The objective of present study is to determine which antipsychotic medication is highly effective in a real-world clinical setting considering adjuvant pharmacotherapy over a 10-year follow-up period.

**Methods:**

A total of 2300 patients with schizophrenia were recruited at the Seoul National University Hospital, and participants received amisulpride, aripiprazole, clozapine, haloperidol, olanzapine, paliperidone, quetiapine, risperidone, ziprasidone for up to 10-years. Time-to-discontinuation of antipsychotic medications was analyzed using Kaplan-Meier survival analyses. Group differences were compared using log-rank tests.

**Results:**

The most frequently used drugs were Risperidone, Aripiprazole, Olanzapine in the antipsychotic drug, Valproate, Lamotrigine, Topiramate in the anticonvulsant agents, Escitalopram, Sertraline, Milnacipran in antidepressants, Lorazepam, Clonazepam, Zolpidem in anxiolytics or sedatives, Propranolol, Benztropine, Trihexyphenidyl in antiparkinson drugs. About half of the patients discontinued taking antipsychotics before 1.5 years. Clozapine showed significantly longer time to discontinuations compared to other antipsychotic drugs. Aripiprazole also showed a lower incidence of discontinuation except for Clozapine and Olanzapine.

**Discussion:**

Clozapine was found to be the most effective antipsychotics in terms of time to discontinuations. Aripiprazole is the most highly recommended 1st line antipsychotics.

